# Glucagon‐Like Peptide‐1 Receptor Agonism and Suicide Risk: Evidence From Mendelian Randomization

**DOI:** 10.1161/JAHA.124.035948

**Published:** 2024-06-15

**Authors:** Sizheng Steven Zhao, Stephen Burgess, Dipender Gill

**Affiliations:** ^1^ Centre for Musculoskeletal Research, School of Biological Sciences, Faculty of Biological Medicine and Health The University of Manchester, Manchester Academic Health Science Centre Manchester UK; ^2^ British Heart Foundation Cardiovascular Epidemiology Unit, Department of Public Health and Primary Care University of Cambridge Cambridge UK; ^3^ Heart and Lung Research Institute, University of Cambridge Cambridge UK; ^4^ Medical Research Council Biostatistics Unit University of Cambridge Cambridge UK; ^5^ Department of Epidemiology and Biostatistics, Imperial College London London UK

**Keywords:** diabetes, glucagon‐like peptide‐1 receptor antagonist, obesity, suicide, weight loss, Epidemiology, Mental Health, Genetics

Glucagon‐like peptide‐1 receptor agonists (GLP1Ra) have proven efficacious for treating type 2 diabetes, obesity, and cardiovascular disease.^1^ However, suicidal thoughts and self‐injury have been reported following treatment with semaglutide, leading to investigations by the European Medicines Agency, and the Medicines and Healthcare Products Regulatory Agency in the United Kingdom. It remains unclear whether these potential adverse events are attributable to semaglutide, the GLP1Ra drug class, the underlying conditions, or other factors. A large observational study found that semaglutide use was associated with lower risk of incident and recurrent suicidal ideation, compared with other antiobesity or antidiabetes medications.^2^ However, it is difficult to draw causal inference from traditional epidemiologic associations because of the potential for residual confounding and reverse causation. For this reason, it is important to triangulate evidence using methods that make orthogonal assumptions. Here, we applied Mendelian randomization (MR) to investigate whether there is human genetic evidence for a causal effect of GLP1Ra on suicide risk. This paradigm uses randomly allocated genetic variants as instrumental variables for studying the effect of GLP1Ra, and is therefore less vulnerable to the environmental confounding and reverse causation bias that can hinder causal inference in traditional epidemiologic designs.

We used single‐nucleotide polymorphisms as instrumental variables for studying lifelong GLP1Ra effects. To proxy the antiobesity effects of GLP1Ra, we selected minimally correlated (*r* ^2^<0.1) single‐nucleotide polymorphisms from within the *GLP1R* gene that were associated (*P*<5×10^−5^, to account for multiple testing given the number of variants in the gene region) with body mass index (BMI) in a genome‐wide association study of 806 834 individuals.^3^ We used BMI as a biomarker for GLP1R perturbation given the recognized effect of GLP1Ra on reducing body weight.^1^ To mimic the antidiabetic effects of GLP1Ra, we also performed analyses that used glycated hemoglobin (n=344 182) and type 2 diabetes liability (242 283 cases and 1 569 730 controls) as biomarkers of the exposure, using the same statistical approach for selecting the instrument variants as for BMI. It is important to consider both the antiobesity and antidiabetic effects of GLP1Ra, as these likely occur through distinct mechanisms.^4^

Genetic association data for suicide attempt (defined as self‐injurious behaviors with an intent to die) and suicide death were taken from a genome‐wide association study meta‐analysis of 43 871 cases (ascertained by interviews, self‐report, or *International Classification of Diseases* [*ICD*] codes) and 915 025 controls.^5^ We repeated analyses using subsets of the outcome data that were European ancestry only (43 871 cases and 915 025 controls) or excluding the UK Biobank (41 438 cases and 580 259 controls). MR analyses were performed using the inverse‐variance weighted method accounting for weak linkage disequilibrium introduced by design. Where possible, we used the MR‐Egger method to assess for directional pleiotropy. This analysis used publicly available summary statistics from studies that had obtained ethical approval. Data are accessible via citations provided.

We identified 2 single‐nucleotide polymorphisms to instrument GLP1Ra effects through BMI, 2 through glycated hemoglobin, and 6 through type 2 diabetes liability (F statistic range, 17–77). We found no strong evidence in support of an association between genetically proxied GLP1Ra and suicide risk, when considering antiobesity (odds ratio, 0.32 per 4.8‐kg/m^2^ reduction in BMI [95% CI, 0.08–1.25]; *P*=0.10) or antidiabetic effects (Figure). Only analyses considering GLP1Ra through reduced type 2 diabetes liability had enough single‐nucleotide polymorphisms for MR‐Egger; there was no evidence of directional pleiotropy (*P* for intercept=0.50). Although nonsignificant, the estimate using BMI as the biomarker is in the opposite direction to the reported association with suicide risk.

**Figure 1:**
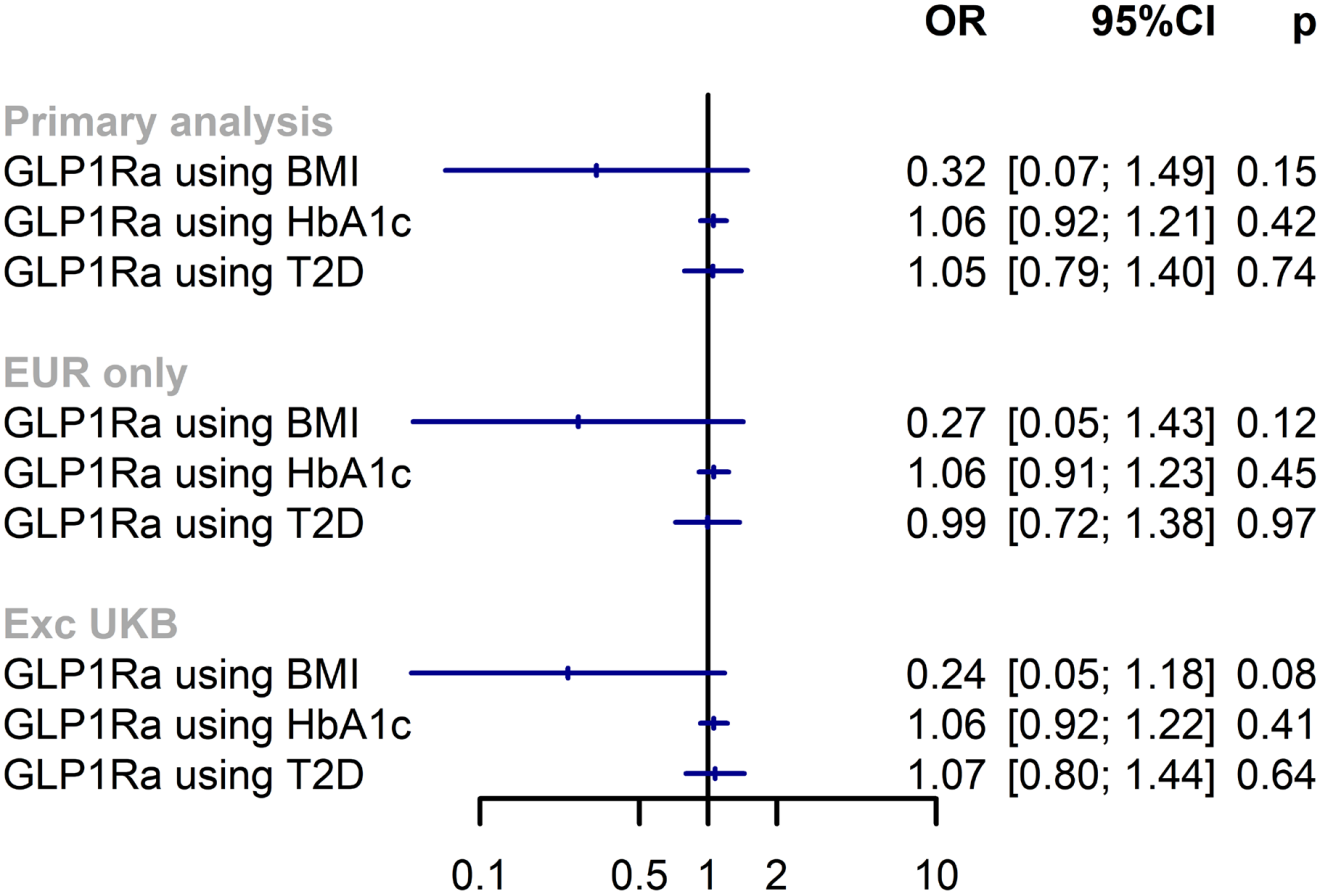
Mendelian randomization analysis results for the association between genetically predicted exposure levels and suicide risk. Estimates shown as per unit reduction in body mass index (BMI; per 4.8‐kg/m^2^ reduction), glycated hemoglobin (HbA1c; per 6.75‐mmol/mol or 1.09% reduction), type 2 diabetes (T2D; per 63% reduction in odds of T2D). Each estimate uses genetic variants in the *GLP1R* gene region, and estimates differ according to the trait used to select variants and scale estimates. Genetic instruments for BMI (PubMed identifier 30239722), HbA1c (www.nealelab.is), and T2D (PubMed identifier 38374256) were selected from within the *GLP1R* gene (build 37; chromosome 6: 39016574–39055519). EUR indicates Europe; Exc, excluding; GLP1Ra, glucagon‐like peptide‐1 receptor agonist; OR, odds ratio; and UKB, UK Biobank.

These findings are in contrast with pharmacovigilance reports of suicide ideation following semaglutide treatment, which may be explained by chance, reporting bias, or potential off‐target effects. The discrepancy may also be explained by differences in the exposure under study: the current genetic associations pertain to small long‐term perturbations in GLP1R signaling, which may differ from therapeutic agonism resulting in larger effects at a discrete point in later life. That is, it remains possible, although not supported by the current evidence, that dramatic short‐term weight loss following initiation of GLP1Ra drugs can increase suicide risk. The genetic analysis showed no evidence for a harmful effect on suicide risk of long‐term perturbation of this pathway.

Our results are concordant with results from a large real‐world study of semaglutide versus other antiobesity and antidiabetic drugs using the TrinetX data source^2^, namely that GLP1Ra is not associated with increased risk. The current triangulation is important because of inherent limitations of pharmacoepidemiologic studies, including residual confounding (eg, large amounts of missing BMI data) and reverse causation (eg, awareness of suicide risk channels such patients away from semaglutide, or improve suicide risk management). As with all MR analyses, the findings are limited by the potential for genetic confounding through unknown pleiotropic effects of the genetic variants used as instruments. These findings alone should not be used to guide clinical practice, but instead inform further research into the link between GLP1Ra and suicide risk. In conclusion, this MR analysis found no strong evidence supporting a potential causal effect of GLP1Ra on increased suicide risk.

## Sources of Funding

Dr Zhao is supported by a National Institute for Health Research Clinical Lectureship and works in centers supported by Versus Arthritis (grant Nos. 21173, 21754, and 21755). Dr Burgess is supported by the Wellcome Trust (225790/Z/22/Z) and the UK Research and Innovation Medical Research Council (MC_UU_00002/7, MC_UU_00040/01). Dr Gill is supported by the British Heart Foundation Research Centre of Excellence (RE/18/4/34215) at Imperial College London.

## Disclosures

None.
